# Effects of BDNF polymorphism and physical activity on episodic memory in the elderly: a cross sectional study

**DOI:** 10.1186/s11556-015-0159-2

**Published:** 2015-12-29

**Authors:** Anne Canivet, Cédric T. Albinet, Nathalie André, Jean Pylouster, Montserrat Rodríguez-Ballesteros, Alain Kitzis, Michel Audiffren

**Affiliations:** Université de Poitiers, Centre de Recherches sur la Cognition et l’Apprentissage, CNRS UMR 7295, Poitiers, France; Institut National Universitaire Champollion, Place Verdun, 81000 Albi, France; Université de Poitiers, CHU de Poitiers, Laboratoire CiMoTheMA – EA 3808 Groupe « Génétique des maladies rares », Poitiers, France; Université de Poitiers, Maison des Sciences de l’Homme et de la Société, CNRS USR 3565, Poitiers, France

**Keywords:** Physical activity, Aging, BDNF Val66Met polymorphism, Episodic memory, Cognition

## Abstract

**Background:**

The brain-derived neurotrophic factor (BDNF) concentration is highest in the hippocampus compared with that in other brain structures and affects episodic memory, a cognitive function that is impaired in older adults. According to the neurotrophic hypothesis, BDNF released during physical activity enhances brain plasticity and consequently brain health. However, even if the physical activity level is involved in the secretion of neurotrophin, this protein is also under the control of a specific gene. The aim of the present study was to examine the effect of the interaction between physical activity and *BDNF Val66Met* (rs6265), a genetic polymorphism, on episodic memory.

**Methods:**

Two hundred and five volunteers aged 55 and older with a Mini Mental State Examination score ≥ 24 participated in this study. Four groups of participants were established according to their physical activity level and polymorphism BDNF profile (Active Val homozygous, Inactive Val homozygous, Active Met carriers, Inactive Met carriers). Episodic memory was evaluated based on the delayed recall of the Logical Memory test of the MEM III battery.

**Results:**

As expected, the physical activity level interacted with BDNF polymorphism to affect episodic memory performance (*p* < .05). The active Val homozygous participants significantly outperformed the active Met carriers and inactive Val homozygous participants.

**Conclusion:**

This study clearly demonstrates an interaction between physical activity and *BDNF Val66Met* polymorphism that affects episodic memory in the elderly and confirms that physical activity contributes to the neurotrophic mechanism implicated in cognitive health. The interaction shows that only participants with Val/Val polymorphism benefited from physical activity.

## Background

Old age is often accompanied by functional and structural changes in the central nervous system. In normal aging, the decrease in the hippocampal volume is generally associated with episodic memory declines [1]. However, the cognitive performances of older adults significantly differ [2]. As age increases, brain resources decrease, and the influence of genetics on cognition becomes increasingly apparent. Genetic variations can explain differences in an individual’s general cognitive ability [3, 4], and the heritability of cognition increases with age [5], from approximately 30 % in childhood to as much as 80 % in adulthood [6].

In humans, a single nucleotide polymorphism (SNP) of the brain-derived neurotrophic factor (BDNF) gene, named *BDNF Val66Met* gene polymorphism or rs6265 SNP, causes a valine (Val) to methionine (Met) substitution at codon 66, which reduces the secretion and distribution of BDNF in the brain [7] to affect episodic memory functioning [8–10] and reduce hippocampal volume [10–12]. The Met allele exerts its effect by impacting intracellular trafficking and the activity-dependent secretion of BDNF. It is associated with reduced neurogenesis and weak cognitive performances. However, other studies have shown that the relationship between the Met allele and cognitive performances is not clearly established [13–15]. A meta-analysis has demonstrated that SNP rs6265 is not associated with hippocampal volume in healthy individuals [16]. Moreover, Erickson et al. [17] showed that Val/Val carriers aged 65 outperformed Met carriers of the same age on an executive functioning task, whereas contradictory results were observed at an average age of 75. Similarly, recent evidence suggests that the functions of the fronto-striatal circuits are more efficient in elderly BDNF Met-allele carriers than in individuals who are homozygous for Val [17–21]. Cognitive processes assessed using event-related potentials (ERPs) seem to vary according to the isoform of the gene [21, 22]. Taken together, these results suggest that the *BDNF Val66Met* polymorphism differently affects various cognitive processes. Currently, the beneficial effect of Val/Val homozygosity on brain structure and brain functioning compared with Met carriers is controversial.

BDNF – a member of the nerve growth factor family – plays an important role in neurogenesis and is implicated in several molecular processes in the central nervous system [23]. In the brain, the expression level of BDNF is highest in the hippocampus, a key region of neural plasticity and adult neurogenesis [24, 25], and this factor is well-known to play an important role in learning and long-term memory [26].

Studies have demonstrated that regular physical activity (PA) is associated with gray matter volume increases in the hippocampus [27] and better memory performance [28]. Study showed that PA increases hippocampal volume, which is implicated in episodic memory [29]. According to these authors, the relationship between exercise and brain health could be explained by the neurotrophic hypothesis, which involves the release of BDNF during exercise [30–32].

In fact, animal studies have shown that the level of BDNF increases in response to exercise [33, 34], and a high level of BDNF subsequently promotes neurogenesis, synapse plasticity, neuronal cell survival and arborization [23, 35]. In humans, the level of BDNF was found to increase after acute exercise [36, 37] or after a 16-week multimodal exercise program in older adults [38]. Thus, an increased concentration of serum BDNF is considered one of the primary molecular pathways by which exercise may improve cognition. PA is shown to increase hippocampal volume and improve episodic memory performance and affects the BDNF level, whereas *BDNF Val66Met* impacts BDNF availability, neuronal survival and morphology and alters neuronal functioning [7, 39]. Therefore, we herein investigated the effect of the interaction between *BDNF Val66Met* polymorphism and PA on episodic memory performance because both these factors are related to BDNF production.

Only four studies highlighted an interaction between *BDNF Val66Met* polymorphism and PA on cognitive performance in older adults, and the results were mixed. A first study of community elders aged 65 years and older showed that the cognitive performances of inactive participants are associated with a decrease in the general cognitive performance as a function of the number of Met alleles in the polymorphism [40]. In a second study of participants aged 60 years and older with mild cognitive impairment, only the BDNF-Met genotype group subjected to a PA program showed a significant increase in the peripheral BDNF level but no differences in general cognition [41]. A third study, in which the participants were 30 to 54 years of age, showed that a high level of PA compensated for the weak performances in working memory of Met carriers but did not significantly affect the Val homozygous individuals [42]. Because these middle-aged participants exhibit less genetic heritability than older individuals [4], extrapolating these findings to older people is difficult. The fourth study showed that only Val homozygous participants benefited from PA, as evidenced by larger hippocampal grey matter and temporal lobe volumes, whereas higher levels of PA were associated with smaller temporal lobe volumes in Met carriers [43]. In these four studies, the BDNF genotype appears to modulate the effects of physical exercise on level of BDNF, cognition and/or brain volume, but the direction of the interaction remains unclear. Testing several alternative hypotheses would be appropriate in order to clarify the direction of this interaction, which was the purpose of the present study.

According to a first hypothesis, if the Met allele negatively affects intercellular trafficking and activity-dependent secretion and PA increases the hippocampal BDNF level, the episodic memory of Met carriers should benefit more from PA than that of Val/Val homozygous individuals. In other words, active Met carriers should perform better in episodic memory tasks than inactive Met carriers, whereas PA should not affect individuals who are homozygous for Val. According to a second hypothesis and in line with Brown’s work [43], PA should optimize the different molecular pathways implicated in cognition and magnify the effect of the Val allele. In other words, only the BDNF-Val homozygous individuals should benefit from the positive effects of PA on episodic memory. The main objective of this study was to validate one of these two hypotheses.

## Methods

### Study

Data were collected from the “PRAUSE” survey conducted in Poitou-Charentes, France, from 2011 to 2013. Four hundred and sixty-six retired volunteers aged 55 years and older (mean age = 75.72; SD = 9.84) were included in the survey. Nonnative French speakers were excluded from participation. The survey was administered at home and in three sessions, with durations of 1.5 to 2 h each. A battery of cognitive tests and questionnaires were administered during these three sessions, and buccal swabs were taken during session 1. The 466 subjects did not all participate in the three sessions.

### Participants

**Fig. 1:**
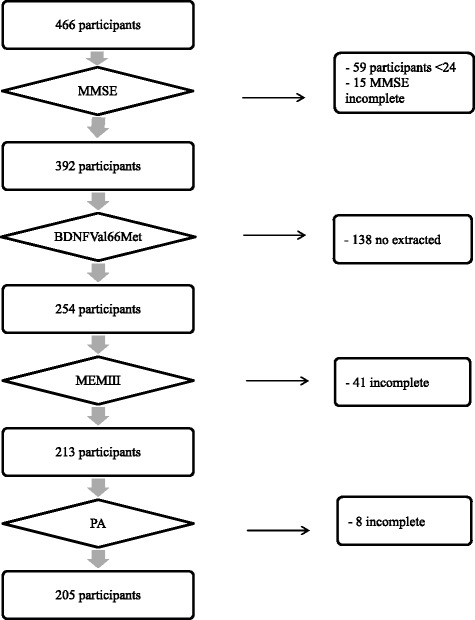
Flow chart describing the selection process of participants. Note: MMSE = Mini Mental State Examination; BDNF Val66Met = Brain derived neurotrophic factor gene polymorphism; MEMIII = Logical Memory test of the MEM III battery; PA = Level of physical activity

Only 205 participants (mean age = 72.72; SD = 9.16) of the 466 volunteers included in the study completed all tests and questionnaires required to verify the hypotheses mentioned in the introduction (see Fig. 1).

All participants provided written informed consent to participate to this survey, which was approved by two national ethics comities: (1) the survey received the “general interest and statistical quality” label from the “Conseil National de l’Information Statistique” (CNIS) [French National Council of Statistical Information] (Visa n°2012X907RG); (2) the survey also received authorization n°1593815 from the “Commission Nationale de l'Informatique et des Libertés” (CNIL) [French National Commission on Informatics and Liberty] (deliberation n°2012-375).

### Cognitive assessments

#### Mini mental state examination

General cognition was evaluated based on the Mini Mental State Examination during the first session, and cognitive impairment (exclusion criterion) was defined as a score below 24 [44].

### Logical memory test II

The Delayed Score of the Logical Memory II subtest of the Wechsler Memory Scale, revised version (WMS-III) [45, 46], was used to assess episodic memory performance. Only story B was read to the participants. Participants listened to the story twice. Immediately after each reading, they were asked to verbally recall as many items as possible about the story. The participants were asked to respond to other questionnaires for twenty minutes, and they were then asked to recall all remembered items of the story without any additional listening. The performance in this task was defined as the total number of correctly recalled items about the story during the delayed recall test. This delayed recall score (max. = 25) is considered an index of episodic memory. We used this test because it is a validated evaluation of episodic memory used worldwide, which offers standardized measures easily comparable with other studies.

### Genotyping

DNA was extracted from buccal cells using the QIAamp DNA Blood Mini Kit (QIAGEN Group) according to the protocol supplied by the manufacturer. SNPs were genotyped using polymerase chain reaction and restriction fragment length polymorphism (PCR-RFLP) analysis. The BDNF polymorphism Val66Met (rs6265) was amplified by PCR using the forward primer 5′-GCCTACCCAGGTGTGCGG-3′ and the reverse fluorescent primer 5′-FAM-GAGGAGGCTCCAAAGGCAC-3′. The PCR products were digested with the restriction enzyme Hsp92II (Promega Corporation) and resolved by capillary electrophoresis in an ABI PRISM 3130 Genetic Analyzer (Life technologies).

Genetic data were analyzed using a dominance model such that Met carriers were combined into a single group because the Val/Met and Met/Met genotypes have been associated with decreased cognitive performance compared with Val-Val genotype [8–10].

### Physical activity

#### The NASA/JSC physical activity scale

During the first session, all participants were asked to rate their regular weekly physical practice on a score from 0 to 7 with the NASA/JSC Physical Activity Scale [47] to identify their level of PA. We used these data to determine the participants’ PA levels in case they left the study after the first session. This questionnaire determines the level of PA less accurately than the subsequent questionnaire that was completed in session 2. It was used to include a maximum of participants and to solve the problem of missing data. The participants concerned by this classification method were considered active if their NASA/JSC physical activity score was strictly higher than 3. To increase the likelihood of observing a significant effect of PA on cognitive performance, we selected only the active participants with a body mass index less than 35 (10 participants); being overweight is generally related to impaired cognitive function [48]. Participants were classified as inactive if their NASA/JSC physical activity score was strictly lower than 3 (127 participants were in this category). Thirty-nine participants scored at level 3 were considered not classifiable because knowing if they practice above or below the recommendations of the World Health Organization (WHO), described hereafter, was impossible.

### The Historical Leisure Activity Questionnaire (HLAQ)

During the second session, the level of current PA was evaluated with the Historical Leisure Activity Questionnaire (HLAQ) [49]. This validated questionnaire was used to assess the history of PA weighted by their relative intensity. Participants were asked to report the frequency, type, intensity, and hours of PA performed during the present year. Using the Compendium of Physical Activities Tracking Guide 2011 [50], we obtained a specific metabolic equivalent (MET) for each PA. According to the HLAQ data and the compendium, we calculated the average energy expenditure (Mets-h/week) for each participant. According to WHO recommendations, we classified the participants above 7.5 METs-h/week in the active group and those below 7.5 METs-h/week in the inactive group.

### Groups constitution

We established 4 groups of participants according to their level of PA (above and below 7.5 METs-h/week, from WHO recommendations) and polymorphism BDNF profile (Met Carriers vs. Val Homozygous) (see Table 1).

**Table 1:** Characteristics of the participants

	Active		Inactive		Total or Average	Effects of PA and BDNF
GROUPS	Val/Val	Met carriers	Val/Val	Met carriers		
Participants (N)	55	48	63	39	205	
Age (SD)	69.69 (7.70)	70.07 (7.70)	77.27 (9.42)	72.93 (9.55)	72.72 (9.16)	PA*
Gender (M/F)	24/31	31/17	17/46	16/23	88/117	PA*, BDNF*
MMSE (SD)	28.35 (1.42)	28.10 (1.56)	27.57 (1.84)	28.36 (1.56)	28.05 (1.64)	NS
Depression score (SD)	6.83 (5.15)	5.85 (4.96)	9.61 (5.37)	8.71 (5.97)	7.81 (5.51)	NS
Education Level (SD)	10.87 (3.24)	10.85 (3.69)	9.69 (3.61)	10.97 (3.94)	10.57 (3.65)	NS
Socioeconomic level (SD)	2439.31 (941.49)	2954.60 (1644.08)	1985.84 (1214.15)	2334.74 (1065.70)	2405.7 (1283.9)	PA*, BDNF*
Hour/week of PA (SD)	10.23 (8.10)	9.31 (6.72)	0.35 (0.61)	0.23 (0.49)	5.68 (7.32)	PA*
Mets-h/week of PA (SD)	52.88 (45.09)	50 (48.35)	1.39 (2.32)	0.83 (1.73)	29.66 (42.93)	PA*

PA* significant main effect of PA, BDNF* significant main effect of BDNF polymorphism, *NS* no effect of PA and BDNF

### The geriatric depression scale (GDS)

The geriatric depression scale (GDS) was used to assess the depression level of participants [51, 52] because several studies showed an interaction between BDNF polymorphism and PA that affected the depression level [53, 54].

### The education level

The education level was measured based on the number of years of formal education, from the first year of elementary school to the third year of a Ph.D. degree (1–20 years). The education level is well known to contribute to the cognitive reserve [55, 56] and strongly influences cognitive performance in older adults.

### The socioeconomic level

The socioeconomic level was assessed from current monthly participants’ income. The scale consisted of 12 classes of monthly income: less than 500 €/month; 500–749 €/month; 750–999 €/month; 1000–1499 €/month; 1500–1999 €/month; 2000–2499 €/month; 2500–2999 €/month; 3000–3499 €/month; 3500–4499 €/month; 4500–5999 €/month; 6000–7499 €/month; more than 7500 €/month. In order to calculate the mean monthly income for each group, we took into account the highest value of the class selected by each individual. A maximal monthly income of 8000 € was assigned for the highest class.

### Statistical analysis

To examine the interaction between BDNF polymorphism and PA that affects episodic memory performance, we first tested the normality of our data distribution using the Lilliefors test. Episodic memory performances were normally distributed (*p* < .01). Thus, we conducted an analysis of variance (ANOVA) on the delayed score of the logical memory test with PA level (active vs. inactive) and BDNF polymorphism (Met carriers vs. Val homozygous) as between-subjects factors. For significant results, mean comparisons were performed using Bonferroni corrections for multiple comparisons. We then conducted an analysis of covariance (ANCOVA) with age, gender and socioeconomic level as covariates. These three variables have been added as covariates because they are significantly associated to PA and BDNF polymorphism in our sample of participants (see Table 1). The two groups of inactive participants were significantly older and socioeconomically weaker than the two groups of active participants. The number of inactive men was significantly lower than the number of inactive women and the number of Met carrier women was significantly lower than the number of Val homozygous women. The Met carriers had a higher socioeconomic level than Val homozygous.

## Results

**Fig. 2:**
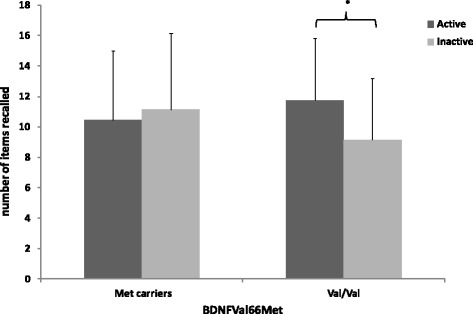
Interaction between BDNF Polymorphism (Met carriers vs. Val/Val) and Physical Activity (active vs inactive) on Episodic Memory Performance. Errors bars represent standard deviation. *p < .01

Our population was divided into 3 allele frequency groups: 10 Met homozygous carriers, 77 Val/Met carriers and 118 Val homozygous carriers, close to the Caucasian breakdown [7]. The allelic frequency was estimated based on the Hardy Weinberg Equilibrium using the khi^2^ test. The distribution of genotypes in the sample did not differ from the Hardy–Weinberg Equilibrium (*p* = 0.57). The ANOVA showed that PA or BDNF polymorphism did not affect episodic memory performance, whereas a significant interaction between PA and BDNF polymorphism did significantly affect episodic memory performance: *F*(1, 201) = 7.06, *p* = .01, ηp^2^ = 0.034 (see Fig. 2). Post-hoc analyses (Bonferroni test) showed that the Inactive and Active Val homozygous carriers significantly differed (*p* < .01), whereas the difference between Inactive and Active Met carriers did not reach significance. The results of the ANCOVA revealed that the BDNF polymorphism × PA interaction remained significant for the episodic memory performance: *F*(1, 198) = 6.05, *p* = .015, ηp^2^ = 0.03. Post-hoc analyses also confirmed the significant difference between Inactive and Active Val homozygous carriers (*p* < .01).

## Discussion

The present study examined the effect of the interaction between PA and *BDNF Val66Met* polymorphism on episodic memory in the elderly. The identification of the genetic background that influences one’s cognition abilities is a challenging task. Physical exercise can mediate the increase of brain BDNF concentrations as an illustration of the gene-environment relationship. Thus, this research aimed to explain the combined influence of genetic polymorphism and one environmental factor (PA). Two opposite hypotheses that considered this interaction were formulated. According to the first hypothesis, PA might boost episodic memory performance in Met carriers because PA increases the level of hippocampal BDNF, which is thought to be deficient in this population, whereas PA should not affect BDNF-Val homozygous carriers. According to the second hypothesis, PA should magnify the effect of the Val allele, resulting in positive effect of PA on episodic memory performance for BDNF-Val homozygous participants only. As expected, we observed a significant interaction between PA and BDNF polymorphism on the delayed score of the logical memory test. First, this interaction shows that BDNF polymorphism modulates the relationship between PA and episodic memory performance, strongly suggesting that PA and BDNF share a common mechanism that influences episodic memory in older adults. More precisely, the direction of this interaction shows that regular PA is associated with better episodic memory only for Val homozygous participants. This result converges with the findings of Brown et al. [43], who showed that only active, Val-homozygous participants benefited from PA and exhibited a larger hippocampal grey matter volume. Clearly, the results of the present study favor the second hypothesis. Specifically, exercise-induced BDNF secretion is more efficient in Val homozygous than in Met carriers.

Since the publication of Egan et al. [7], the investigation of genetic associations between BDNF and cognition has produced mixed results, and these discrepancies can be attributed to the ethnic group, age of the participants or choice of controlled variables. Among the controlled variables influencing cognition, life habits, such as PA or the mental health and education level of the participants, are rarely controlled, but these factors are known to influence the cognitive performance of older people. The strength of the present study is that it highlighted the interaction between polymorphism and PA, a life habit recognized to benefit brain health. We showed that PA interacts with the *BDNF Val66Met* to affect the episodic memory performance. Thus, controlling the PA level is important when examining the putative impact of *BDNF Val66Met* on cognition in the elderly.

From a phylogenetic perspective, examining the link between the BDNF gene and PA would be interesting. According to a phylogenetic hypothesis [57], the necessity to be physically active is probably programmed into our genes and has remained relatively stable for the past 10 000 years [58]. We know that our Homo sapiens ancestors had to be highly active for their survival and that the Val homozygote BDNF, which is the wild-type genotype, secretes proteins more effectively. The advantage of the Val homozygous genotype for brain BNDF secretion, hippocampal volume and episodic memory performance could be magnified in active humans. The physical ability to find food and to use it as a source of energy for the brain could act in a cooperative association with mechanisms underlying cognitive functions [59] and increase the adapted survival strategies. We can hypothesize that long ago, these two features, PA and BDNF Val/Val, linked in order to be efficient.

If the wild-type, Val/Val, seems to optimize the effects of BDNF secretion for people practicing regular PA, the subsistence of the Met allele (homozygous and heterozygous) in the population must be linked to both the evolution of the species and its life habits. Our results may also be interpreted in light of the possibility that sedentary lifestyle is a phylogenetically new phenomenon, with negative consequences on health, brain and behavior, whereas the human genome, a product of a very long evolutionary process, was built for active organisms fighting for their survival. In other words, the different genetic variations of some genes, like *BDNF Val66Met*, remain in the population because the survival of sedentary people is being facilitated by the development of human technology.

Moreover, some studies have demonstrated that Met allele carriers are more protected during aging than the Val/Val group, especially with respect to cognitive functions that involve the prefrontal cortex (PFC), such as executive functions [19, 20, 60]. Specifically, Erickson et al. [42] showed that this advantage was magnified by PA for Met carriers. These results appear to contradict our results and other results obtained in several studies assessing the relationship between cognition and *BDNF Val66Met* [14, 15]. However, Getzmann et al. [22] consider that the effect of BDNF polymorphism may differ depending on the brain areas supporting the different cognitive functions. Future studies should more precisely examine the cognitive functions that are negatively affected by specific iso-forms of the *BDNF Val66Met* polymorphism using more selective cognitive evaluations; i.e., a large battery of two or three cognitive tests per cognitive function that declines with age, mainly the speed of information processing, episodic memory and executive functions. Currently, PA has been shown to enhance cognitive vitality and facilitate cognitive tasks that rely on the hippocampus and the prefrontal cortex. Thus, future research should explore whether the effect of PA on various domains of cognitive performance depends on the *BDNF Val66Met* polymorphism.

Moreover, these findings could be attributed to an alternative explanation. According to Egan et al. [7], Pezawas et al. [61] and Hariri et al. [62], Met carriers are subject to a stronger diminution of resources with age than Val homozygous individuals. Even if they practice PA, which increases the level of BDNF [37], reaching the threshold of protein that counterbalances the effects of aging is more difficult for BDNF Met carriers. Consequently, the episodic memory performances of Met carriers do not change, irrespective of PA. By contrast, PA may help Val homozygous individuals to attain the optimal threshold of BDNF concentration and outperform their inactive Val homozygous counterparts.

Four main limitations can be noted. First, this study was cross-sectional, and the measure of physical activity was based on the participants’ self-reports. Therefore, reporting may have been subject of bias due to the subjective perception of participants about their PA level. However, the HLAQ questionnaire and the NASA/JSC Physical Activity Scale are widely recognized and well-validated instruments. Second, this cross-sectional study design limits the ability to interpret these results as a causal relationship between physical activity and cognition; conversely, better cognitive functioning may result in individuals being more physically active. Third, the brain BDNF levels were not assessed through lumbar puncture because this methodology was not compatible with the PRAUSE protocol, and therefore the interaction between PA and BDNF polymorphism is only associative. Fourth, a haplogroup (group of genes transmitted together) may exert protective or compensative effects on certain alterations in the secretion of proteins linked to the polymorphism genes. For example, several studies have demonstrated that for the Met allele, the Asian-type population [63] seems to be protected from cognitive aging. This ethnic influence was not considered in our study, which was conducted in France on a mostly Caucasian population. Future studies should adopt a randomized controlled trial approach to determine in a causal link for the benefit of PA training programs to BDNF Val homozygous carriers and various types of population in order to generalize the results.

## Conclusions

To conclude, we observed a significant interaction between PA and BDNF polymorphism that affected the delayed score of the logical memory test. This result shows that the association between PA and episodic memory was mediated by BDNF polymorphism because PA increased the episodic memory performance only in Val-Val homozygous participants.

## Abbreviations

BDNF: Brain-derived neurotrophic factor
HLAQ: Historical Leisure Activity Questionnaire
MEMIII: Logical Memory test of the MEM III battery
MMSE: Mini Mental State Examination
PA: Physical activity
SNP: Single nucleotide polymorphism
WHO: World Health Organization
